# Computational fluid dynamics modeling of coronary artery blood flow using OpenFOAM: Validation with the food and drug administration benchmark nozzle model

**DOI:** 10.3233/XST-230239

**Published:** 2024-08-16

**Authors:** Sajid Ali, Chien-Yi Ho, Chen-Chia Yang, Szu-Hsien Chou, Zhen-Ye Chen, Wei-Chien Huang, Tzu-Ching Shih

**Affiliations:** a Graduate Institute of Biomedical Sciences, China Medical University, Taichung, Taiwan; b Department of Family Medicine, China Medical University Hsinchu Hospital, Hsinchu, Taiwan; c Department of Internal Medicine, Division of Cardiovascular Medicine, China Medical University Hsinchu Hospital, Hsinchu, Taiwan; d Department of Radiology, China Medical University Hsinchu Hospital, Hsinchu, Taiwan; e Center for Molecular Medicine, China Medical University Hospital, Taichung, Taiwan; f Department of Medical Laboratory Science and Biotechnology, Asia University, Taichung, Taiwan; g Department of Biomedical Imaging and Radiological Science, China Medical University, Taichung, Taiwan

**Keywords:** Computational fluid dynamics (CFD), coronary computed tomography angiography, food and drug administration (FDA) benchmark nozzle model, fractional flow reserve (FFR)

## Abstract

Cardiovascular disease (CVD), a global health concern, particularly coronary artery disease (CAD), poses a significant threat to well-being. Seeking safer and cost-effective diagnostic alternatives to invasive coronary angiography, noninvasive coronary computed tomography angiography (CCTA) gains prominence. This study employed OpenFOAM, an open-source Computational Fluid Dynamics (CFD) software, to analyze hemodynamic parameters in coronary arteries with serial stenoses. Patient-specific three-dimensional (3D) models from CCTA images offer insights into hemodynamic changes. OpenFOAM breaks away from traditional commercial software, validated against the FDA benchmark nozzle model for reliability. Applying this refined methodology to seventeen coronary arteries across nine patients, the study evaluates parameters like fractional flow reserve computed tomography simulation (FFR_CTS_), fluid velocity, and wall shear stress (WSS) over time. Findings include FFR_CTS_ values exceeding 0.8 for grade 0 stenosis and falling below 0.5 for grade 5 stenosis. Central velocity remains nearly constant for grade 1 stenosis but increases 3.4-fold for grade 5 stenosis. This research innovates by utilizing OpenFOAM, departing from previous reliance on commercial software. Combining qualitative stenosis grading with quantitative FFR_CTS_ and velocity measurements offers a more comprehensive assessment of coronary artery conditions. The study introduces 3D renderings of wall shear stress distribution across stenosis grades, providing an intuitive visualization of hemodynamic changes for valuable insights into coronary stenosis diagnosis.

## 1 Introduction

Cardiovascular disease (CVD) is a leading cause of mortality and morbidity and a primary contributor to reduced quality of life worldwide. CVD has a lifetime risk exceeding 60% and causes a substantial economic burden for countries in the form of direct healthcare expenses, reduced worker productivity, and informal public aftercare [1, 2]. In the past decade, the global mortality rate of CVD increased to 12.5%. Among all types of CVD, coronary artery disease (CAD) is the second most prevalent [2]. Several risk factors for CAD worldwide include overweight and obesity, high blood lipids, tobacco use, high blood pressure, low physical activity, diabetes, family history, and poor nutrition [3]. Health-care providers throughout the United States have encouraged people to adopt a healthier lifestyle, which has been shown to reduce the risk of CVD and other diseases. Despite this guidance, nearly half of all Americans are affected by CVD [1]. However, the mortality rates of CVD in high-income countries like the United States have been reduced by access to advanced medical tools for diagnosing and treating the disease. In low-income and middle-income countries, which do not have the resources to implement such tools in their health-care systems, the number of CVD-related deaths continues to increase [2, 4, 5]. If health-care systems are to become more sustainable, they must increase patient access to medical facilities that offer early diagnosis and treatment, reduce medical costs, and enhance the effectiveness of the various patient care pathways [6].

The primary cause of CAD is stenosis of the coronary artery, which is often caused by atherosclerosis. Invasive coronary angiography with fractional flow reserve (FFR) is the current gold standard for determining the clinical relevance of stenotic lesions [7, 8]. This procedure involves the insertion of a catheter and guide wire being into the radial or femoral artery. The catheter is then carefully guided into the ascending aorta, the point of origin for the coronary arteries. Here, a sensitive pressure wire is employed to measure the pressure ratio between locations beyond and closer to the stenosis. This particular procedure necessitates the administration of a drug, often adenosine, to the patient in order to induce hyperemia [9, 10]. However, one study reported that 28% of patients who undergo invasive coronary angiography with FFR experience adverse reactions to adenosine. Although severe adverse effects are rare, documented reactions to adenosine include bronchospasm, tachyarrhythmia, and cardiac arrest [11–13]. Moreover, the radiation dose that patients receive during invasive coronary angiography is substantially higher than that received during coronary computed tomographic angiography (CCTA), an alternative procedure for diagnosing stenosis [14].

Computational fluid dynamics (CFD) has recently been adopted to predict the distribution of hemodynamic parameters in the human circulatory system by using anatomical information gathered through coronary CT angiography. This noninvasive method has become a key means of simulating coronary artery blood flow and predicting the functional severity of stenoses. Simulating blood flow provides insight into the intricate hemodynamics of the cardiovascular system [11, 15–17]. Fluid–structure interaction and rigid wall models are used in CFD to estimate arterial pressure; such numerical simulation results have been compared to those of invasive intravascular ultrasonography [18].

This study analyzed the distribution of hemodynamic parameters in the coronary arteries of patients with mild to severe serial stenoses by using the open-source software OpenFOAM. Patient-specific three-dimensional (3D) models of coronary arterial networks were reconstructed from images captured through noninvasive CCTA. we employed a numerical approach based on the finite volume method to solve the Navier-Stokes equations and the continuity equation for transient flow problems. The finite volume method discretizes the fluid domain into a finite number of control volumes and solves the equations governing fluid flow for each of these volumes over small time steps. Hemodynamic parameters, encompassing essential variables such as pressure, velocity, and wall shear stress, were meticulously extracted and quantified through a rigorous analysis of the simulated flow field. Therefore, the comprehensive assessment allowed for a thorough understanding of the dynamic interactions within the fluid system under investigation.

## 2 Methods

### 2.1 The food and drug administration (FDA) benchmark nozzle model

**Fig. 1:**
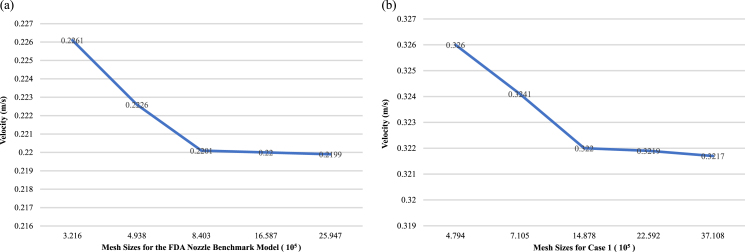
Grid independent test conducted within two models with the inlet velocity set at 0.2 m/s and the outlet boundary condition imposed as zero pressure: (a) the FDA nozzle benchmark model, and (b) the patient-specific model (Case 1).

The US Food and Drug Administration (FDA) benchmark nozzle model is a widely used and standardized method for validating blood flow simulations of realistic coronary artery geometries, as shown in Fig. 1(a). The present study generated unstructured volumetric meshes with boundary layers and uniform node spacing with the ICEM CFD software package (ANSYS, Inc., Canonsburg, PA, United States) to ensure the reproducibility of the results. A total of five meshes were generated for the FDA nozzle benchmark and the patient-specific model Case 1. These meshes consisted of 224,500 to 2,594,657 elements and 479,425 to 3,710,825 nodes, respectively, using varying grid sizes. Furthermore, the maximum speed disparities at the exit plane for both the FDA nozzle benchmark and the patient-specific model were determined to be under 0.7%. This suggests that the simulation results were not affected by the choice of grid size and were consistent with what was expected based on the physical behavior. Our study highlights the potential of the FDA benchmark nozzle as an effective tool for evaluating and validating blood flow simulations, as demonstrated by the consistent and reliable results obtained across different grid sizes. These findings provide valuable guidance for the design and implementation of future studies in this field, ultimately contributing to improved accuracy and reliability of blood flow simulations in the medical community.

**Fig. 2:**
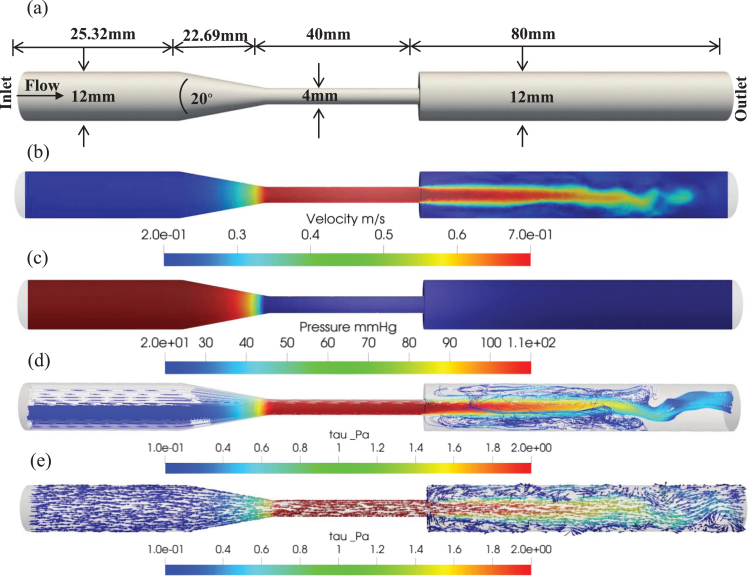
(a) The FDA benchmark nozzle model: geometric length and conical geometry with rapid diameter specifications, (b) flow velocity distribution, (c) pressure distribution, (d) streamline distribution, and (e) vector flow distribution.

The resulting fluid velocity and pressure profiles were monitored, as demonstrated in Figs. 2(b) and 2(c). A narrowing of the lumen can lead to an increase in flow velocity at the throat of the FDA benchmark nozzle model, resulting in low shear stress in the upstream region, high shear stress in the throat region, and disturbed flow in the form of directionally oscillatory shear stress in the downstream region, as illustrated in Figs. 2(d) and 2(e). These findings provide valuable insights into the complex flow dynamics associated with stenosed coronary arteries and have major implications for the diagnosis and treatment of CVDs.

In all simulations, a maximum Courant number of 0.5 and a constant time step of *Δ*t = 1×10^–5^ s were used. The velocity profile at the inlet was parabolic, and the initial velocity and pressure were both set to zero. The no-slip boundary condition, which assumes zero velocity of the fluid at the solid boundary, was applied to the walls of the model. Additionally, the pressure at the outlet was set to zero, while blood density was set to 1,060 kg/m^3^, and the blood viscosity was 4 cP.

### 2.2 Coronary computed tomography angiography acquisition

CCTA images were acquired using a 64-slice CT scanner (Aquilion 64, Toshiba Medical Systems, Japan) with 64 simultaneous detector rows; a 320 mm scan field of view with a slice thickness of 0.5 mm was used. The CT images were taken captured in the Digital Imaging and Communications in Medicine (DICOM) images format, reconstructed using the Toshiba Vitrea Workstation (Toshiba Medical Systems Corporation. Aquilion 64) with stereolithography (STL) file format, and subsequently loaded into Mimics software (Mimics Research 21.0). Image segmentation was performed manually using Mimics software to generate 3D geometric models of the objects of interest. Segmentation involved the selection and labeling pixels or voxels within the images that corresponded to the desired object or region and then refinement for obtaining a precise representation of the object’s boundaries. The segmented images were then used to generate 3D geometric models in Mimics. This manual segmentation approach enabled the operator to precisely control and adjust the segmentation parameters to ensure the accuracy and fidelity of the models. The resulting 3D geometric models were saved in the STL file format.

### 2.3 Geometric models of coronary arteries

The 3D coronary artery models, which comprised an aorta, both coronary arteries, and their branches, were imported as STL files into ANSYS SpaceClaim. To reduce computational time, a method described by Kwon et al. (2018) was used in which the aorta and branches was removed from the models, leaving only the coronary arteries. The estimated difference in FFR values between model simulations with and without the aorta was less than 2% [19]. In our study, we initiated the process by meticulously refining the surface meshes of the reconstructed 3D coronary arteries, ensuring the elimination of any irregularities with the indispensable aid of ANSYS SpaceClaim.

The incorporation of extensions in our model serves a crucial purpose, aiming to ensure the comprehensive development of flow both prior to entry and subsequent to exit from the actual geometric structure. This strategic inclusion plays a pivotal role in maintaining the integrity of the inlet and outlet boundary conditions within the artery model. An extension of insufficient length poses the risk of the flow not attaining a fully developed parabolic profile upon entering or leaving the authentic geometry. This inadequacy may lead to undesirable perturbations at the boundaries, compromising the accuracy of the simulation. On the contrary, an excessively lengthy extension unnecessarily escalates the computational mesh and simulation runtime. Achieving an optimal balance is thus imperative in selecting the extension length.

To uphold a continuous and unobstructed blood flow within our model, we judiciously introduced cylindrical extensions at both the inlet and outlet points of the arteries. These locations were carefully chosen where the blood flow had already attained its fully developed state. The strategic incorporation of these extensions played a crucial role in safeguarding uninterrupted flow dynamics at the simulation model boundaries. The determination of the cylindrical extension length was guided by the semiempirical formula Le/D≈0.06*Re*, specifically applicable to laminar flow conditions [20]. This formula ensured a calculated and precise length tailored to the laminar flow dynamics of our system.

For accurate simulation of blood flow in the left and right coronary arteries, a meticulous approach was taken. Two outlets and one inlet patch were created for the left coronary artery, while a single inlet and outlet patch were designated for the right coronary artery. This meticulous patch design aimed to mirror the physiological characteristics of blood flow in each artery, contributing to the overall fidelity of our simulation model.

Delaunay triangulation was applied using ANSYS ICEM CFD to discretize the complex 3D coronary artery models into unstructured tetrahedral meshes [21]. Compared with hexahedral meshes, tetrahedral meshes are more memory efficient and have lower computational costs for complex geometries while producing similar results. Therefore, unstructured tetrahedral meshing is an efficient solution for accurately capturing the flow physics for complex 3D coronary artery models.

In the simulations utilized for the final analysis, more than 10 million grid points were employed to strike a balance between computational time and numerical precision. The resulting geometry was then imported into OpenFOAM to simulate blood flow. Gravity and phase change effects were not used. Blood viscosity, density, wall condition, maximum Courant number, and the number of time steps were the same as those used in the FDA benchmark nozzle model. The inlet blood velocity was set at 0.2 m/s for all simulations [22].

## 3 Results

The present study performed CCTA on nine patients to evaluate 18 arteries. However, one artery was excluded from the study because its image was of inadequate quality; 17 arteries were thus included in the final simulation. Among the nine patients, 8 RCAs and nine left main coronary arteries were observed, which emerged from the fusion of the left circumflex and left anterior descending arteries. Table 1 provides the age and sex of the patients, branch of the coronary artery examined, and numbers of elements and nodes in the simulated model.

**Table 1:** Distribution of demographic data and artery characteristics for 9 patients, including element numbers for each branch, node number, total element number, and computational time in simulations

Subjects	Age	Gender	Branch	Lengths (mm)	Elements number	Nodes number	Total element	Computational
					for branch (×10^5^)	(×10^5^)	number (×10^5^)	time (hours)*
Case 1	67	Male	LAD	137.49	3.13	9.57	28.09776	267
			LCx	97.12
Case 2	––	––	RCA	98.51	2.79	5.53	38.89692	370
Case 3	70	Male	LAD	144.128	3.73	8.69	14.18663	135
			LCx	108.62
Case 4	––	––	RCA	88.35	2.41	4.58	27.62442	263
Case 5	36	Female	LAD	140.67	3.30	8.41	23.22094	221
			LCx	93.35
Case 6	51	Male	LAD	105.27	3.43	8.47	31.46997	299
			LCx	86.074
Case 7	––	––	RCA	147.3	4.76	31.48	31.46992	299
Case 8	28	Female	LAD	142.27	3.42	10.77	10.40473	99
			LCx	79.94
Case 9	––	––	RCA	85.26	2.03	3.40	26.76311	255
Case 10	68	Female	LAD	114.66	3.56	7.76	10.54769	100
			LCx	99.24
Case 11	––	––	RCA	99.36	2.28	3.92	16.47762	157
Case 12	49	Female	LAD	93.861	4.64	7.31	10.85332	103
			LCx	67.89
Case 13	––	––	RCA	89.36	2.07	3.48	30.28896	288
Case 14	61	Male	LAD	115.42	3.05	9.59	16.03558	153
			LCx	94.62
Case 15	––	–––	RCA	95.56	2.59	3.06	28.33497	270
Case 16	50	Male	LAD	126.299	3.82	8.99	20.87993	199
			LCx	95.12
Case 17	–––	Male	RCA	98.01	2.11	3.40	22.59217	215

*The computational time was determined using an Intel Core™ i5-1035G1 CPU operating at 1.19 GHz, coupled with 12.0 GB of RAM.

### 3.1 Grid independence analysis in computational blood flow simulations for stenosed coronary arteries

**Fig. 3:**
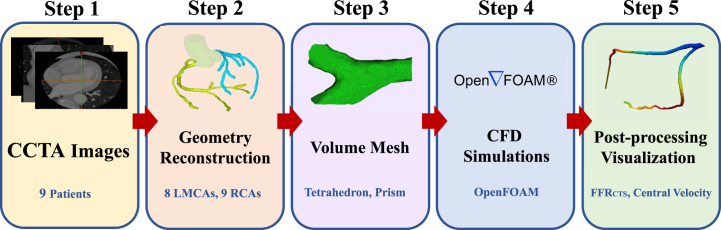
Flowchart for simulation.

We performed a comprehensive grid independence analysis on 3D coronary artery models, utilizing data gathered from various cases, including the illustrative Case 1 depicted in Fig. 1(b). This analysis involved conducting a mesh independence study to assess how computational grid size affects the accuracy and reliability of blood flow simulations of a stenosed coronary artery. The maximum velocity and pressure drops were compared for each of the five grid sizes. Although the use of larger grids led to a slight increase in both maximum velocity and pressure drop, the differences in the results obtained using various grid sizes were small, suggesting that the flow modeling was reliable and accurate. Taken together, these findings suggest that the computational methods used in the present study are robust and capable of generating accurate simulations of blood flow through a stenosed coronary artery, regardless of the size of the computational grid used. These results have crucial implications for the design and implementation of future studies modeling and researching the complex dynamics of blood flow, as shown in Fig. 3.

### 3.2 Integrating FFR_CTS_ and central velocity for comprehensive diagnostic insights

**Fig. 4:**
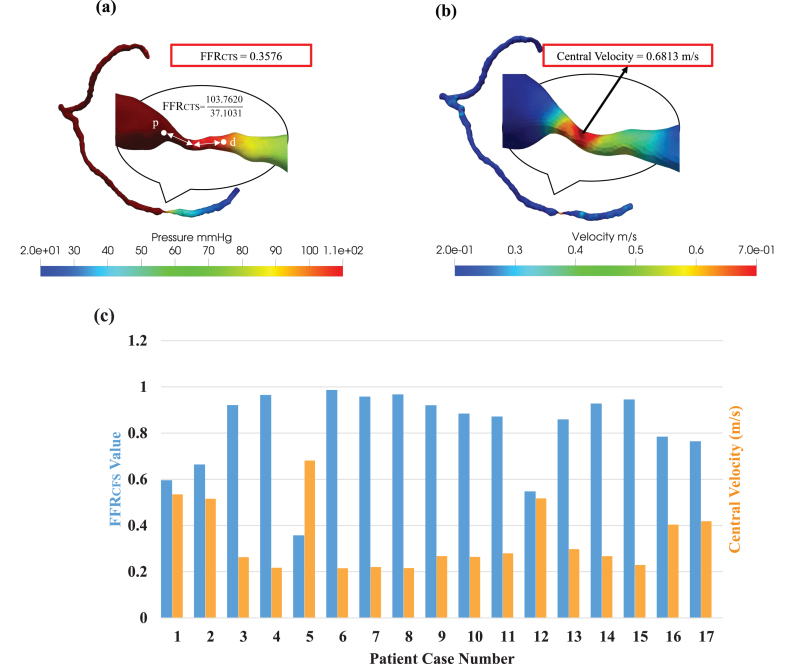
(a) Fractional flow reserve derived from computational tomography simulation (FFR_CTS_) and (b) central velocity mapping in Severe Stenosis patient. (c) probing the complex relationship between FFR_CTS_ and central velocity in various clinical scenarios.

We conducted blood pressure measurements at two specific locations: one closer to the stenosis (proximal) and the other farther away (distal) as shown in Fig. 4(a). To ascertain the fractional flow reserve through computed tomography simulation (FFR_CTS_ value), we divided the pressure at the stenosis by the pressure at the proximal site. Furthermore, we employed CT images to simulate blood flow within the narrowed section of each artery and assess the distribution of blood flow velocity precisely at the center of the stenosis, as depicted in Fig. 4(b). Our approach offers a comprehensive and quantitative evaluation of the hemodynamic parameters, which plays a critical role in accurately characterizing the severity of coronary stenoses. In our diverse clinical scenarios (Fig. 4(c)), we consistently observed an inverse relationship between FFR_CTS_ and central velocity across all cases. This inverse correlation was especially pronounced in Case 5, where the FFR_CTS_ value measured 0.3576, while the central velocity registered at 0.6813 m/s. Furthermore, this velocity was 3.4 times higher than the inlet velocity of 0.2 m/s, clearly highlighting the significant impact of severe stenosis on blood flow velocity. The estimation of FFR_CTS_ and central velocity serves as a valuable tool for clinicians in assessing stenosis severity and its potential consequences on blood flow, enabling more informed diagnostic decisions [23, 24]. In addition to quantitative data, encompassing FFR_CTS_ values and central velocity measurements, we also gathered qualitative data by assessing the stenosis grades of our study participants. These evaluations were performed by medical professionals employing CCTA. Stenosis grades serve as indicators of the extent of vascular narrowing or blockage and are categorized on a scale from 0 to 5. Moreover, a grade of 0 denotes the absence of stenosis (equivalent to 0% stenosis), while a grade of 5 signifies complete occlusion (or 100% stenosis). Our analysis revealed varying degrees of stenosis among the participants, with cases distributed as follows: grade 0 (*n* = 3), grade 1 (*n* = 2), grade 2 (*n* = 6), grade 3 (*n* = 2), grade 4 (*n* = 3), and grade 5 (*n* = 1), as detailed in Table 2.

**Table 2:** Simulation results of FFR_CTS_ and central velocity measurements for all cases (*n* = 17)

	Physician Diagnosis		CFD Simulations	
	(Qualitative)		(Quantitative)	
# Cases	Stenosis Grade	Stenosis Level	FFR_CTS_	Central Velocity (m/s)
3	Grade 0	No (0%)	0.973±0.009	0.216±0.001
2	Grade 1	Minimal (1–24%)	0.952±0.006	0.224±0.004
6	Grade 2	Mild (25–49%)	0.898±0.026	0.486±0.124
2	Grade 3	Moderate (50–69%)	0.775±0.009	0.411±0.007
3	Grade 4	Severe (70–99%)	0.602±0.116	0.781±0.048
1	Grade 5	Total (100%)	0.358±0	0.681±0

FFR_CTS_ fractional flow reserve in computed tomography simulation. # Cases (*n* = 17): combined with the left main, *n* = 9 and right coronary arteries, *n* = 8.

### 3.3 Integrated hemodynamic metrics FFR_CTS_ and central velocity unveil comprehensive insights for informed clinical decision-making

**Fig. 5:**
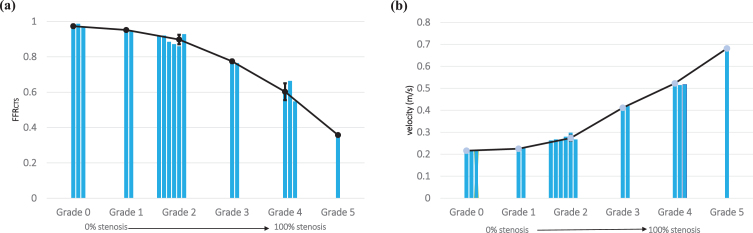
This study meticulously examines the significant contributions of two vital hemodynamic parameters, (a) FFR_CTS_ value and (b) central velocity, in effectively forecasting the extent of stenosis severity.

The FFR_CTS_ value emerges as a valuable metric for gauging the severity of stenoses across different grades. In grade 0 cases, FFR values exceeded 0.8, while conversely, in the case of grade 5 (representing severe to total occlusion), the FFR_CTS_ value fell below 0.5, as illustrated in Fig. 5(a). Likewise, in grade 1 instances, central velocity closely mirrored the inlet velocity, but in the grade 5 scenario, central velocity surged to 3.4 times the inlet velocity, as demonstrated in Fig. 5(b). We also found that our CFD findings exhibited inconsistencies for grade 2 cases, underscoring the limitations of conventional FFR measurement approaches in accurately grading such stenoses. This underscores the paramount importance of employing FFR_CTS_ and central velocity measurements for enhanced precision in assessments. By mixing qualitative stenosis grade data with quantitative FFR_CTS_ and central velocity measurements, clinicians can glean comprehensive insights into the severity and potential ramifications of stenosis on blood flow. Such knowledge serves as an invaluable compass for determining the most suitable treatments or interventions for patients.

### 3.4 Unveiling hemodynamic variations in stenosis: A comprehensive analysis across minimal, moderate, and critical cases

**Fig. 6:**
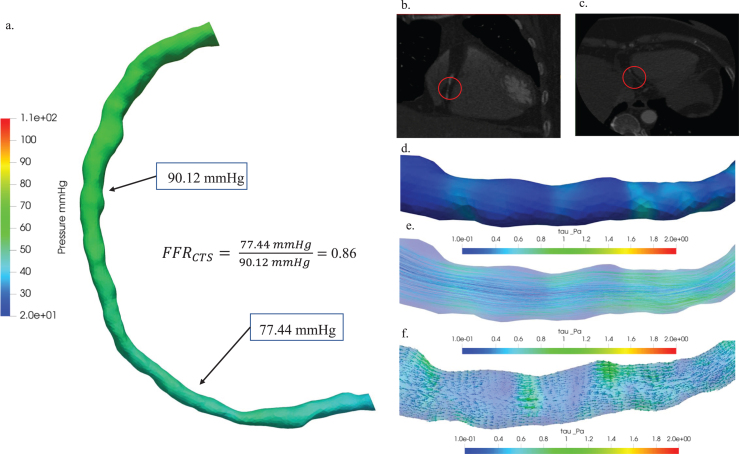
Hemodynamic characterization of stenosis in patient Case 13: (a) A comparative study of pressure distribution and FFR_CTS_ value, (b) 3D visualization of blood vessel CT images: axial view (b) and coronal view (c), plaque locations highlighted with red circles. Hemodynamic visualization of wall shear stress (d), streamline (e), and vector (f).

The classification employed in our analyses enriches the clinical significance by illustrating how hemodynamics vary according to the severity of stenosis. This research comprehensively investigated the hemodynamic implications associated with stenosis across three distinct patient cases: Cases 13, 1, and 5. Case 13 corresponds to a scenario of minimal stenosis, Case 1 is indicative of moderate stenosis, and Case 5 is characterized by a critical stenosis condition. Figure 6 presented a thorough hemodynamic analysis of minimal stenosis in patient Case 13, providing valuable insights into its characterization. The figure showcased key aspects of this analysis: (a) a comparative study involving pressure distribution diagrams and a measured FFR_CTS_ value of 0.86, enabling a nuanced understanding of the hemodynamic significance of the stenosis; (b) the axial view of the 3D visualization of the blood vessel CT image, offered a detailed perspective on the stenotic region; (c) the coronal view of the blood vessel CT image, which enhanced the anatomical context of stenosis by emphasizing plaque locations with red circles; (d) hemodynamic visualizations illustrated the distribution of wall shear stress, a crucial element in comprehending the impact of stenosis on blood flow dynamics; (e) the distribution of streamlines in (e), aided in visualizing blood flow patterns within the stenotic region for a comprehensive understanding of hemodynamic behavior; and (f) the depiction of vectors in (f), added another layer of insight into the intricate hemodynamics associated with the stenosis by representing the direction and magnitude of hemodynamic forces.

**Fig. 7:**
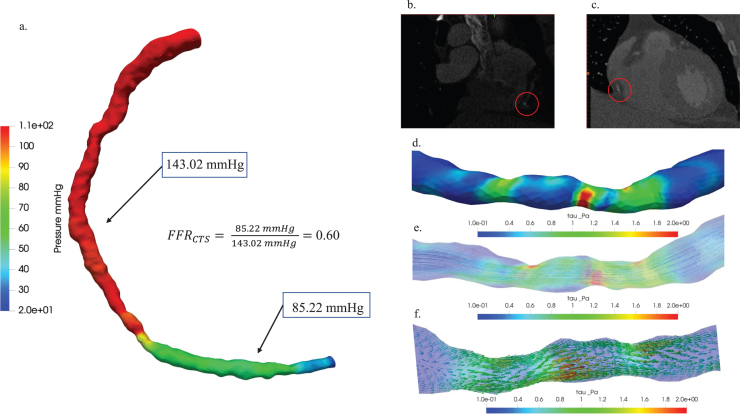
Hemodynamic characterization of stenosis in patient Case 1: (a) A comparative study of pressure distribution and FFR_CTS_ value, (b) 3D visualization of blood vessel CT images: axial view (b) and coronal view (c), plaque locations highlighted with red circles. Hemodynamic visualization of wall shear stress (d), streamline (e), and vector (f).

Moreover, Fig. 7 illustrated the hemodynamic characterization of moderate stenosis in patient Case 1. (a) The study presented a comparison of pressure distribution diagrams and FFR_CTS_ values, providing insights into pressure variations within the stenotic region and the hemodynamic significance of stenosis, with a FFR_CTS_ value of 0.60 as a reference point. (b) The axial view of the blood vessel CT image and (c) the coronal view were visually depicted in 3D, with red circles highlighting plaque locations to emphasize the anatomical context of stenosis. Hemodynamic visualization distributions of (d) wall shear stress, (e) streamline, and (f) vector were also showcased in the past tense, offering a comprehensive overview of the hemodynamic characteristics associated with stenosis in this specific patient case.

**Fig. 8:**
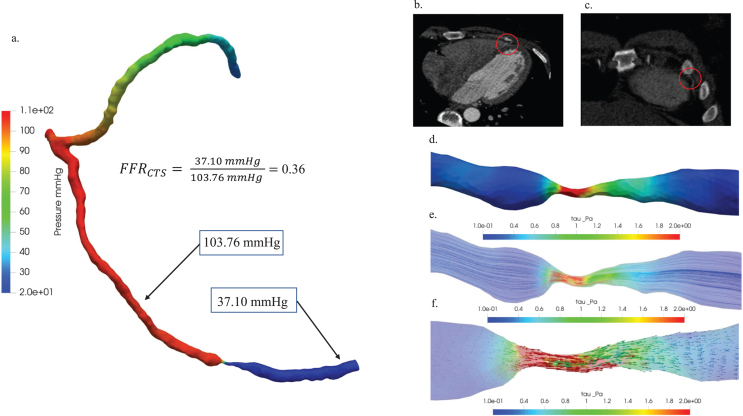
Hemodynamic characterization of stenosis in patient Case 5: (a) A comparative study of pressure distribution and FFR_CTS_ value, (b) 3D visualization of blood vessel CT images: axial view (b) and coronal view (c), plaque locations highlighted with red circles. Hemodynamic visualization of wall shear stress (d), streamline (e), and vector (f).

In Fig. 8, an in-depth hemodynamic characterization of critical stenosis in patient Case 5 was undertaken through a comprehensive analysis. Firstly, a comparative examination of pressure distribution diagrams and FFR_CTS_ values (a) provided valuable insights into pressure variations within the stenotic region, elucidating the hemodynamic significance of the stenosis. The FFR_CTS_ value of 0.36 played a pivotal role as a reference point for this assessment. Further enhancing the visual understanding, the 3D representations of (b) the axial view and (c) the coronal view of the blood vessel CT image were presented, strategically incorporating red circles to highlight plaque locations and underscore the anatomical context of the stenosis. Additionally, (d) wall shear stress, (e) streamline, and (f) vector were meticulously examined to visually represent the hemodynamic distributions associated with stenosis in the context of patient Case 5.

## 4 Discussion

In the present study, one of the largest limitations was the image quality of the Aquilion 64 64-slice CT scanner. This multislice CT system was used with settings of 0.5 mm slice thickness, 64 simultaneous detector rows, and a 320 mm field of view for image acquisition, which may have limited the accuracy of our reconstructions of the 3D coronary artery geometry and thus our calculations of the FFR_CTS_ values. Another limitation was the computational time, which exceeded 1 week for each case on an Intel Core (TM) i5-1035G1 CPU at 1.19 GHz and 12.0 GB of RAM. Computational time is highly influenced by grid size, increasing the grid size increases the computational time. Thus, increasing grid size for greater accuracy would exacerbate the long computation times.

We used CTS to analyze the hemodynamic characteristics of blood flow in coronary arteries with different grades of stenosis. We observed an inverse relationship between FFR_CTS_ and central velocity in all cases, with severe cases exhibiting low FFR_CTS_ values and high central velocity. These findings are consistent with the findings of previous research that has used CFD to analyze coronary artery stenosis [25, 26]. We performed CTS simulations to simulate blood flow in a model of coronary stenosis and observed that the severity of stenosis was associated with a decrease in FFR_CTS_. Similarly, we analyzed the hemodynamic characteristics of blood flow in coronary arteries with stenosis and discovered that the FFR was inversely related to the severity of stenosis [27].

Our results indicate that FFR_CTS_ is a useful metric for assessing the severity of stenoses of various grades. The FFR_CTS_ value was more than 0.8 in cases of normal, minimal or mild stenosis (grade 0–1) and less than 0.8 in cases of moderate, severe or total occlusion (grade 3–5). These findings are consistent with those of previous research that has used FFR to assess the severity of stenosis. For instance, Ntalianis [28] conducted a meta-analysis of studies that used FFR to assess the severity of stenosis and reported that an FFR cutoff of 0.8 had high sensitivity and specificity for predicting ischemia.

In this research endeavor, our objective is to employ a noninvasive technique, specifically Fractional Flow Reserve derived from Computed Tomography (FFR_CTS_), in conjunction with uncomplicated boundary conditions. This approach aims to derive FFR_CTS_ values across three distinct scenarios representing mild, severe, and critical stenosis conditions. To facilitate this, various measurement distances were considered, as detailed in Table 3. Moreover, the empirical FFR_CTS_ measurement distance in actual patient cases amounted to approximately 20 mm. The tabulated data consistently reveals a noteworthy trend: in all scenarios, a reduction in the measurement distance corresponds to an elevation in the FFR_CTS_ value. Conversely, an elongated measurement distance is associated with diminished mean pressure levels at the distal extremity of the stenosis, consequently leading to decreased FFR_CTS_ values. This pattern substantiates the proposition that the FFR value is intricately tied to both the length of the measurement distance and the specific positions at which measurements are taken Table 3 [26].

**Table 3:** Hemodynamic significance assessment: simulated FFR_CTS_ values at different measurement distances in patients

Subjects	Distance (mm)	*Pp (mmHg)	#Pd (mmHg)	FFR _CTS_
Case 13	10	87.5413	81.4709	0.9306
Mild Stenosis	12.5	88.4881	79.9204	0.8907
	15	89.5685	78.5393	0.8769
	17.5	89.6394	78.0613	0.8708
	20	90.1254	77.4496	0.8593
	22.5	91.5255	76.4151	0.8349
	25	91.6315	76.2087	0.8317
	27.5	91.9858	75.9698	0.8256
	30	93.5393	74.9698	0.8015
Case 1	10	140.980	82.6529	0.6097
Severe Stenosis	12.5	141.134	82.9093	0.6198
	15	141.282	83.5494	0.6195
	17.5	142.339	84.5898	0.6171
	20	143.020	85.2219	0.5959
	22.5	144.029	88.8849	0.5943
	25	144.604	89.5858	0.5914
	27.5	145.795	90.3656	0.5875
	30	150.117	91.5225	0.5863
Case 5	10	103.102	40.7155	0.3949
Critical Stenosis	12.5	103.351	39.4670	0.3819
	15	103.409	38.9528	0.3767
	17.5	103.602	38.0011	0.3668
	20	103.762	37.1031	0.3576
	22.5	103.861	36.2824	0.3493
	25	104.595	35.1173	0.3357
	27.5	104.396	34.0243	0.3259
	30	104.328	33.1502	0.3020

*Pp: proximal Pressure #Pd: Distal pressure FFR_CTS_: fractional flow reserve computed tomography simulation.

Finally, we analyzed the distribution of shear stress in the stenotic region and observed high shear stress caused by the narrowing of the lumen. This finding is consistent with that of previous research demonstrating that stenosis can increase shear stress and alter flow patterns in blood vessels, which can affect vascular biology and increase the likelihood of CVD. For instance, Chiu and Chien [29] reviewed the effects of shear stress on endothelial cells and reported that high shear stress can lead to endothelial dysfunction and atherosclerosis.

Our integrated approach makes significant contributions, showcasing three key innovations. Firstly, by employing OpenFOAM CFD tools, an open-source software, we enhance accessibility and cost-effectiveness, fostering broader engagement in cardiovascular hemodynamics research. Secondly, our study introduces Fractional Flow Reserve derived from Computed Tomography (FFR_CTS_) as a non-invasive metric, offering a comprehensive evaluation of coronary stenosis across grades 0 to 5. This expanded spectrum enables nuanced treatment planning, surpassing the traditional focus on stenosis severity. Thirdly, our exploration of wall shear stress (WSS) provides insights into vascular health, identifying regions of high shear stress associated with remodeling and low WSS linked to endothelial dysfunction and atherosclerosis. Integrating these innovations, our study contributes a holistic perspective on coronary hemodynamics, emphasizing accessibility, comprehensive evaluation, and nuanced insights to refine diagnostic and treatment approaches in cardiovascular health. While acknowledging limitations, including computational time and potential image quality constraints, we recognize the need for future refinements and optimizations to further enhance the applicability of our methodology.

The extended exposure to heightened shear stress, especially in specific arterial regions, has emerged as a key contributor to vascular remodeling [30]. Our technique enables the precise identification of areas undergoing elevated shear stress, thereby contributing to arterial dilation and outward remodeling. This well-documented phenomenon acts as a potential precursor to aneurysm development [30], underscoring the significance of our approach in deciphering the mechanisms governing such vascular adaptations. Moreover, exceedingly high shear stress, particularly in regions featuring stenosis or other irregularities, has been linked to hemolysis, leading to the release of hemoglobin and other substances into the bloodstream [31]. Our methodology delivers a detailed mapping of shear stress levels in these critical areas, providing insights into potential implications for vascular health. By unraveling the intricate relationship between shear stress and hemolysis, our approach contributes to a nuanced understanding of hemodynamic factors influencing cardiovascular diseases. The impact of shear stress on platelet activation and aggregation is a critical aspect [32]. Our technique excels in identifying regions characterized by high shear stress and disturbed flow patterns, offering a valuable tool for assessing thrombosis risk. By pinpointing areas prone to platelet activation, our approach lays the groundwork for developing targeted interventions to mitigate the risk of thrombotic events in patients with vascular abnormalities. Highlighting the critical role of shear stress in maintaining endothelial cell function, our approach pays particular attention to aberrant patterns, such as low or oscillatory shear stress. These patterns are known contributors to endothelial dysfunction and the progression of CVD [33]. By elucidating the impact of shear stress on endothelial health, our technique adds a layer of sophistication to understanding the complexities of CVD progression. The clinical implications of our study are substantial. Armed with detailed insights into blood flow patterns and shear stress distribution, clinicians can identify and monitor vulnerable vascular areas. This knowledge, combined with our understanding of shear stress in CVD, opens the door for the development of targeted interventions. From preventive measures to tailored treatments, our technique represents a promising avenue for enhancing patient outcomes in the realm of cardiovascular health.

## 5 Conclusions

This study may provide valuable insight into the hemodynamics of and potential effects of stenosis on coronary arteries. We combined qualitative stenosis grading with quantitative FFR_CTS_ and central velocity measurements to comprehensively assess stenosis severity and its blood flow impact. The consistent inverse relationship between FFR_CTS_ and central velocity highlights their clinical significance. Additionally, our visualization of shear stress distributions and vectors offers crucial insights into arterial wall hemodynamics and potential effects on vascular health. This underscores the importance of utilizing advanced imaging and computational techniques for evaluating coronary stenosis hemodynamics.
